# Phenol Removal Capacity of the Common Duckweed (*Lemna minor* L.) and Six Phenol-Resistant Bacterial Strains From Its Rhizosphere: In Vitro Evaluation at High Phenol Concentrations

**DOI:** 10.3390/plants9050599

**Published:** 2020-05-08

**Authors:** Olga Radulović, Slaviša Stanković, Branka Uzelac, Vojin Tadić, Milana Trifunović-Momčilov, Jelena Lozo, Marija Marković

**Affiliations:** 1 Department of Plant Physiology, Institute for Biological Research “Siniša Stanković”–National Institute of the Republic of Serbia, University of Belgrade, 142 Bulevar Despota Stefana, Belgrade 11060, Serbia; branka@ibiss.bg.ac.rs (B.U.); milanag@ibiss.bg.ac.rs (M.T-M.); marija.nikolic@ibiss.bg.ac.rs (M.M.); 2 Faculty of Biology, University of Belgrade, 16 Studentski Trg, Belgrade 11000, Serbia; slavisas@bio.bg.ac.rs (S.S.); jlozo@bio.bg.ac.rs (J.L.); 3 Mining and Metallurgy Institute Bor, 35 Zeleni Bulevar, Bor 19210, Serbia; vojin.tadic@ibiss.bg.ac.rs

**Keywords:** bacteria, bioremediation, duckweed, phenol, rhizosphere

## Abstract

The main topic of this study is the bioremediation potential of the common duckweed, *Lemna minor* L., and selected rhizospheric bacterial strains in removing phenol from aqueous environments at extremely high initial phenol concentrations. To that end, fluorescence microscopy, MIC tests, biofilm formation, the phenol removal test (4-AAP method), the Salkowski essay, and studies of multiplication rates of sterile and inoculated duckweed in MS medium with phenol (200, 500, 750, and 1000 mg L^−1^) were conducted. Out of seven bacterial strains, six were identified as epiphytes or endophytes that efficiently removed phenol. The phenol removal experiment showed that the bacteria/duckweed system was more efficient during the first 24 h compared to the sterile duckweed control group. At the end of this experiment, almost 90% of the initial phenol concentration was removed by both groups, respectively. The bacteria stimulated the duckweed multiplication even at a high bacterial population density (>10^5^ CFU mL^−1^) over a prolonged period of time (14 days). All bacterial strains were sensitive to all the applied antibiotics and formed biofilms in vitro. The dual bacteria/duckweed system, especially the one containing strain 43-*Hafnia paralvei* C32-106/3, Accession No. MF526939, had a number of characteristics that are advantageous in bioremediation, such as high phenol removal efficiency, biofilm formation, safety (antibiotic sensitivity), and stimulation of duckweed multiplication.

## 1. Introduction

Phenol and its derivatives are used in all fields of industry. However, excessive industrial growth, especially in developing countries, results in the constant influx of phenol into the environment worldwide [1]. Phenol is toxic even at very low concentrations (2 mg L^−1^) and is notoriously hard to eliminate by standard physical and chemical methods [2]. Bioremediation, i.e., the use of the natural ability of some organisms to detoxify their surroundings, is proposed as a welcome alternative, especially in cases when large areas are affected [1,2,3,4,5].

In addition to constant background leaks of phenol from hospitals, households, and factories, catastrophic leaks of >1 ton of phenol are relatively rare, but not uncommon in many countries worldwide (in Sweden—Gothenburg, 1973; S. Korea—the Nakdong River incident, 1991; Vietnam—the Formosa Ha Tinh steel disaster in 2016) [1,2]. Phenol is readily soluble in water and easy to disperse—therefore, in aquatic ecosystems, the local effect of phenol leaks spreads rapidly from the entry site. Phenol can cause severe damage to the aquatic life, plants, and particularly aquatic vertebrates, as well as to humans, even at very low concentrations of less than 0.1 ppm [1,2]. Unintentional chlorination of phenol in water treatment facilities increases the risk to human health since chlorinated phenols are known carcinogens and more stable than non-substituted phenol [2].

The toxic effects of phenol on living cells are non-specific: as a nucleophile, it targets all cellular proteins, causing structural damage and failure of various enzymatic processes. Mitochondria and chloroplasts are the most susceptible cellular organelles to phenol-induced damage [6,7,8]. Many phenolic herbicides and even some naturally occurring phenolic compounds that suppress the growth of competing plant species can disrupt the respiratory chain [7]. Studies of phenol carcinogenicity are inconclusive, but many of its derivatives, like chlorophenols, are known to be mutagenic and carcinogenic, which can be traced back to phenol indirectly interfering with DNA synthesis and reparation by disrupting the synthetic and reparatory enzymes [9,10]. Bacterial resistance to phenol is also non-specific and relies on the chemical properties of their cell walls and membranes. Gram-negative bacteria are long known to be more resistant to phenol than Gram-positive bacteria due to the complexity of their cell wall [9].

In contrast to bacteria and many unicellular fungi that are able to metabolize phenol even at high initial concentrations, the list of multicellular, complex organisms that are resistant to these extremely unfavorable environmental conditions and at the same time capable of detoxifying their surroundings is limited. However, many plants can effectively eliminate various noxious compounds through adsorption, accumulation, or transformation, even under very unfavorable conditions. This process is a subtype of bioremediation referred to as phytoremediation [11,12,13]. Whereas the majority of phytoremediation studies focus on terrestrial plants, the bioremediation potential of aquatic plants is relatively less investigated. Water plants like common water hyacinth (*Euchhornia crassipes*), water lettuce (*Pistia stratiotes*), the reedmace (*Typha latifolia*), and the common reed (*Phragmites australis*) are frequently used in phytoremediation [13,14,15]. However, their application in phenol phytoremediation is often limited by several factors such as geographical distribution and invasiveness, the need for soil, relatively low propagation rates, and propensity for heavy metal removal rather than the removal of organic compounds. Duckweeds (Lemnaceae) are cosmopolitan, non-invasive, floating aquatic plants generally recognized as versatile bioremediation agents. Their bioremediation potential relies on their unique physiology and morphology: instead of the typical stem of higher plants, duckweeds possess a thallus-like body called fronds that reproduces almost exclusively in a vegetative manner [16,17]. This fast reproduction leads to large biomass production, a property that can be utilized in the treatment of wastewaters with high organic or heavy metal content [18,19,20].

Although all duckweeds are known to possess a considerable degree of tolerance to toxic compounds, two particularly wide-spread species, *Lemna minor* and *L. gibba*, are the most commonly studied and shown to be capable of removing various phenolic derivatives from aqueous media. The giant duckweed (*Spirodela polyrhiza*) also received some attention in the recent research, but as an allochthonous species in Europe and due to slow growth compared to *L. minor* or *L. gibba* (almost four-fold slower, according to Ziegler et al.), it is not considered a preferable model organism for the studies of biological removal of phenol [20,21].

As a rule, the bioremediation potential of duckweeds does not rely solely on their own enzymes. The microbial communities of their rhizosphere and the region surrounding their root surface influence the outcome of bioremediation, which is true for other plant species as well [21,22,23,24]. Moreover, the rhizosphere of duckweeds stimulates the growth of autochthonic, non-pathogenic bacterial strains [22]. However, there has been a limited number of efficient phenol-degrading bacterial strains isolated from the rhizosphere of the Lemnaceae family, thus far [25,26,27]. The genetic structure of rhizosphere-associated microbiota varies greatly between different *L. minor* ecotypes and is determined by ecological factors (e.g., climate, geographical region, temperature). However, the presence of phenol-degrading bacteria seems to be one of the few common denominators because the duckweeds, like all other plants, tend to attract the phenol-degrading bacteria with their naturally occurring phenolic exudates [27]. Phenolic compounds are universal signaling molecules of the plants, but in some cases, bacteria have evolved to utilize them as a nutrient source as well [28]. The rhizosphere is only recently being recognized as a valuable source of phenol-degrading bacteria; until recently, the most efficient phenol-degrading bacterial strains (able to remove phenol at initial concentrations as high as 1000 mg L^−1^) were isolated from industrial effluents or polluted soil samples [26].

In this paper, the phenol-eliminating ability of *L. minor* and the bacterial strains isolated from its rhizosphere, as well as some other aspects of their interaction that are beneficial to bioremediation (biofilm formation, antibiotic resistance, interactions with the root, and bacterial effect on multiplication rates of the plants), were examined.

## 2. Results

### 2.1. Characterization of Bacterial Strains (Biofilm Formation and Antibiotic Resistance)

**Figure 1:**
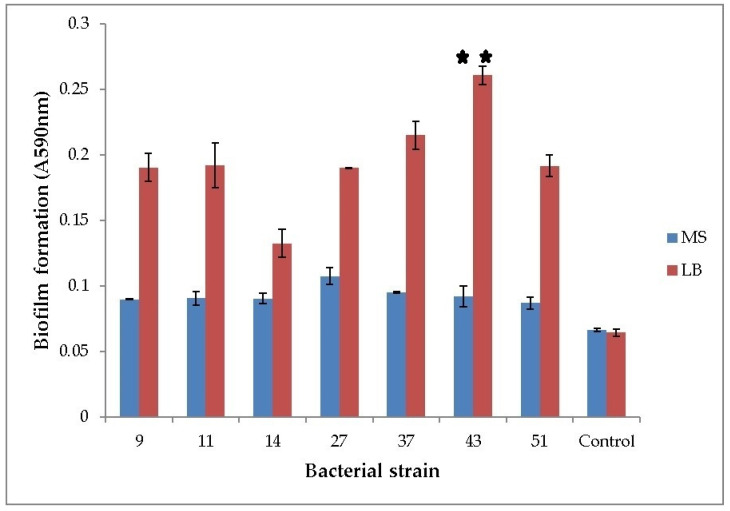
Biofilm formation of phenol-degrading bacteria. Seven bacterial strains were grown at 25 °C in LB and phenol-supplemented MS medium. *Y*-axis represents optical density of biofilm (OD_590nm_). Numbers on the *x*-axis represent bacterial strains: 9—*K. oxytoca*, 11—*Lelliottia*, 14—*K. oxytoca*, 27—*S. marcescens*, 37—*H. alvei*, 43—*H. paralvei*, 51—*S. nematodiphila*. Control is sterile medium (LB or MS with phenol). Data represent the mean ± SE (*n* = 3). Mean with ** is significantly different and signifies the best biofilm formation. One-sample *t*-test, 6 df, *p* < 0.01.

All of the seven tested strains formed biofilms. When grown in LB medium, the ability of *H. alvei* and *H. paralvei* to form a biofilm was approx. 10% and 33% greater compared to the average biofilm formation of the group, respectively. Only the biofilm formation of *H. paralvei* strain 43 was significantly greater compared to the rest of the group (one-sample *t*-test with *p* < 0.01, the mean value with statistical significance annotated with “**”; Figure 1). There was no statistical difference within the group grown in MS medium supplemented with phenol (one-sample *t*-test with *p* < 0.05). Biofilm formation by bacteria grown in LB medium was greater when compared to bacteria grown in MS medium supplemented with phenol (one-way ANOVA test with *p* < 0.05; Figure 1). Results of 16S rDNA sequencing for previously taxonomically undetermined bacterial strains (11 and 14) and of gyrB sequencing for *Serratia* sp. (27) and *Serratia nematodiphila* (51) suggested that strain 11 is *Lelliottia* sp., strain 14 *K. oxytoca*, and both strains 27 and 51 are *S. marcescens*, respectively (Table S1).

Antibiotic resistance is presented as a minimal inhibitory concentration (MIC), i.e., the lowest concentration of the antibiotic suspended in the soft (0.7%) LB agar (in mg L^−1^) that inhibited bacterial growth. All of the tested bacterial strains are susceptible to antibiotics, albeit with different MICs (Table S2).

### 2.2. Salkowski Reagent Test for the IAA Detection in the MS Liquid Medium

The IAA production in the tested bacterial strains was not detected with the Salkowski essay, which implies that the IAA of bacterial origin is not the reason for the increased multiplication rates of duckweed. However, according to Gilbert et al., the Salkowski essay cannot detect ≤5 μg/mL of IAA when no exogenous L-tryptophan is supplemented into the medium (as is the case in this study) [29]. Therefore, it is possible that these strains still produced a very low level of IAA that was not detected by this method.

### 2.3. Fluorescence Microscopy of the Plant Root-Bacteria Interactions

**Figure 2:**
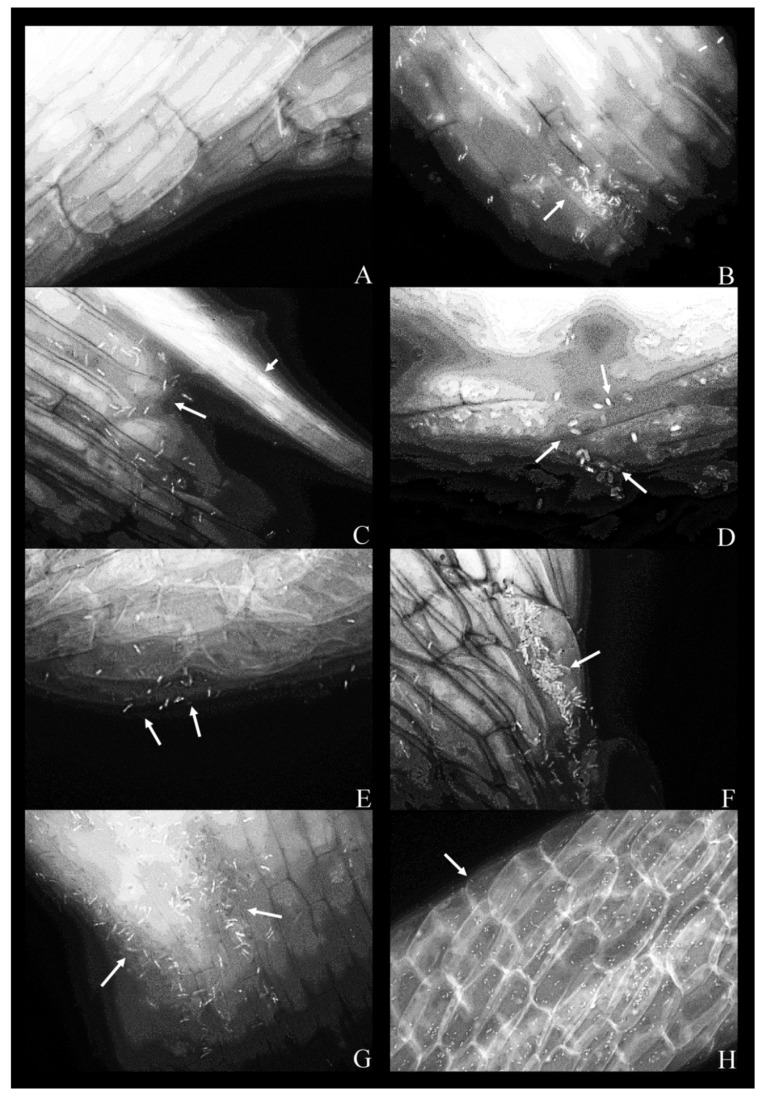
Fluorescent micrographs of duckweed roots in phenol-free MS medium with bacterial strains: (**A**) 9—*Klebsiella oxytoca*; (**B**) 11—*Lelliottia* sp., a bacterial microcolony on the root surface (arrow); (**C**) 14—*K. oxytoca*, bacterial cells on the root surface (arrow) with a vascular bundle (arrowhead); (**D**) 14—*K. oxytoca* (magnified 100×), bacterial cells arranged around a rupture in the root; (**E**) 27—*Serratia marcescens*, representative bacterial cells on the root surface (arrows); (**F**) 37—*H. alvei*, bacterial cells organized in a microcolony (arrow); (**G**) 43—*H. paralvei*, a microcolony; (**H**) 51—*S. nematodiphila* (magnified 40×); bacterial cells are arranged along the lines between the epidermal cells of the root. Magnification: 63×, unless stated otherwise. Arrows: bacterial cells on the root surface. Arrowhead: a vascular bundle.

For conducting the fluorescence microscopy, seven bacterial strains were co-cultivated with duckweed over a period of five days in the phenol-free MS medium. Out of these, six strains attached to the plant root surface (Figure 2B–H), whereas strain 9 (*K. oxytoca*) was not observed (Figure 2A). Bacteria tend to form smaller local aggregations that can be described as microcolonies in the intercellular crevices between neighboring plant root epidermal cells. In the phenol-free MS medium, all strains retained their expected, rod-shaped form. Filter used was FITC: Chroma set 41,001, Excitation 460–500 nm, Emission 510–560 nm (bandpass filter).

**Figure 3:**
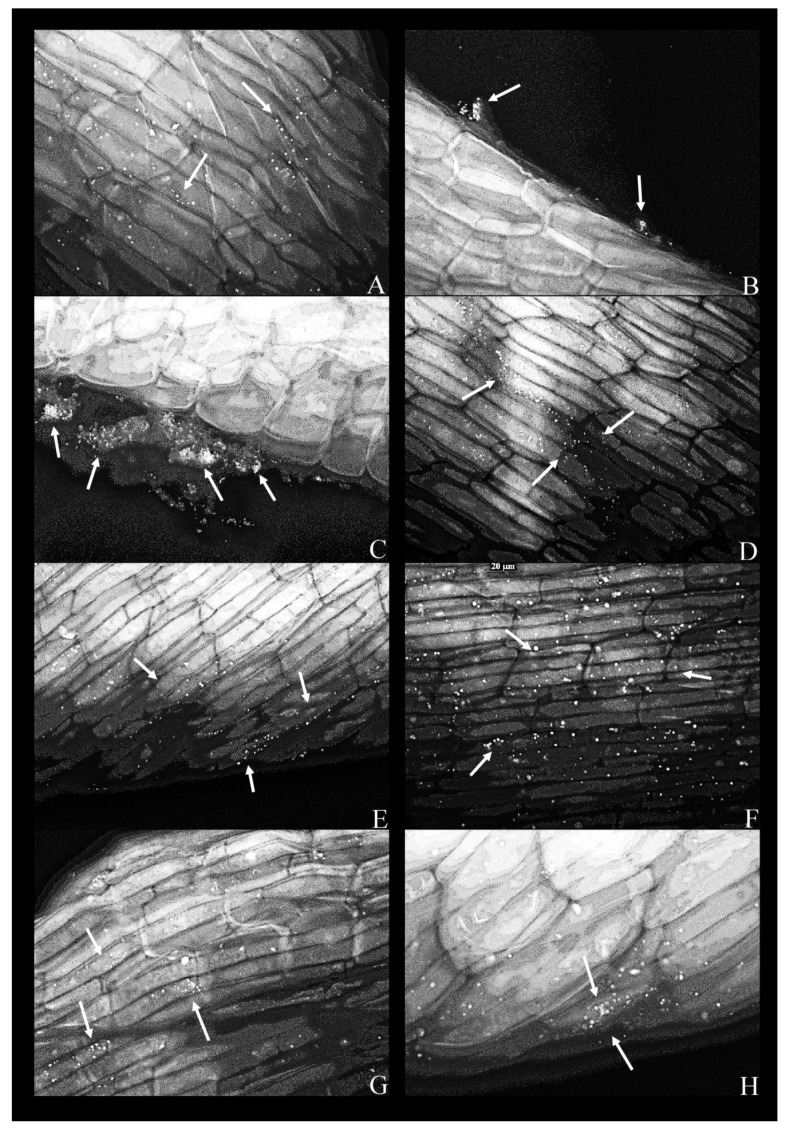
Fluorescent micrographs of duckweed roots in phenol-supplemented (500 mg L^−1^) MS medium with bacterial strains: (**A**) 11—*Lelliottia* sp., bacterial cells scattered on the root surface(arrows); (**B**) 14—*Klebsiella oxytoca*, dense groups of bacterial cells near the root surface (arrows); (**C**) 14—*K. oxytoca* (63×), dense aggregates of bacterial cells (indicated by arrows) near the regions of the root destroyed by pressure (squashing); (**D**) 27—*Serratia marcescens*, bacterial cells organized along the lines between epidermal cells of the root (arrows); (**E**) 37—*Hafnia alvei*, bacterial cells organized on the root surface and along the lines of cell–cell boundaries (arrow); (**F**) 43—*H. paralvei*, bacterial cells on the root surface; (**G**) 51—*S. nematodiphila*, scarce bacterial cells on the root surface; (**H**) 51—*S. nematodiphila*, magnified 63× to show a typical microcolony on the root surface. Magnification: 40×, unless stated otherwise. Arrows: bacterial cells on the root surface.

The same experiment was conducted in the MS supplemented with phenol (200 mg L^−1^). In this case, all strains (Figure 3A–H), with the exception of *K. oxytoca*, strain 9 (not shown), attached to the root surface.

As a rule, a bacterial presence was observed only along the middle portion of the root, but never in the apical or the basal portion (adjacent to the frond).

In the presence of phenol, bacteria were coccoid as opposed to having the conventional rod shape (Figure 3). In the case of *K. oxytoca*, strain 14, dense bacterial aggregates appeared to be “squeezed” out of the plant—these aggregates formed in the proximity of ruptures or cuts in the plant tissue (Figure 3B,C). The same strain was scarce on the root surface in the phenol-free MS (Figure 2C).

**Figure 4:**
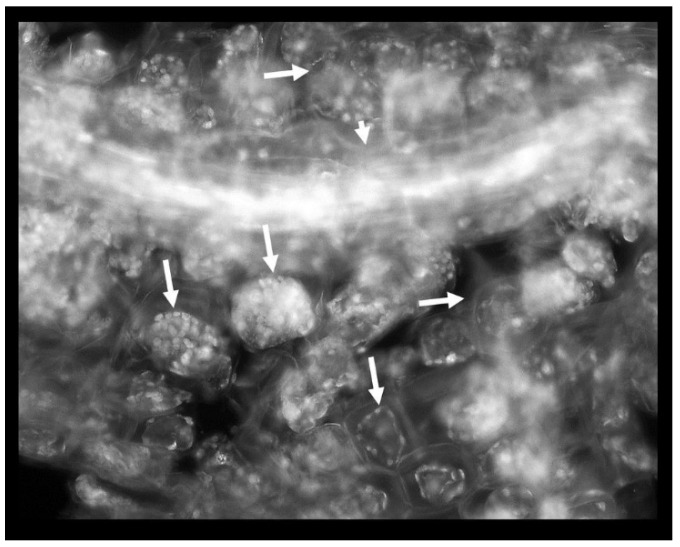
Phenol-induced root damage is represented by the abundance of intensely autofluorescing plastids and extensive plasmolysis (arrows). Short arrow: a vascular bundle.

Phenol was detrimental to the root: the cell walls appeared thicker albeit more brittle (more susceptible to mechanical damage) in the presence of phenol; plastids were abundant and organized in the cell periphery; these plastids were probably non-pigmented leucoplasts and their autofluorescence was stemming from their polysaccharides, lipid or protein content; there were entire regions of the root affected by plasmolysis as evidenced by plasma membrane withdrawal from the cell walls (Figure 4).

### 2.4. Multiplication of the Duckweed Specimens Cultured with Different Bacterial Strains

**Figure 5:**
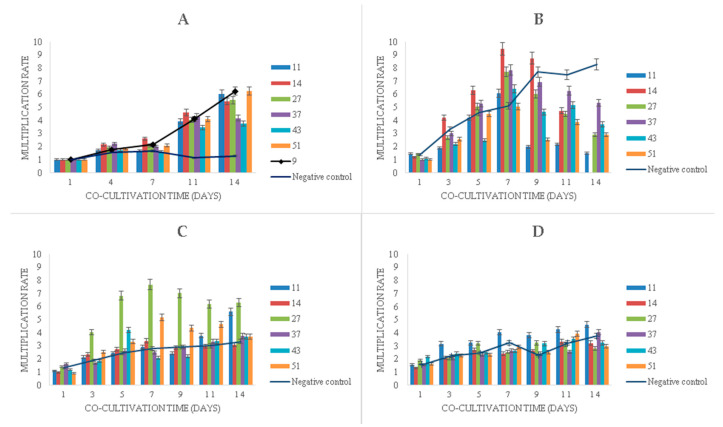
Multiplication rates of duckweed in MS medium: (**A**) without phenol; (**B**) with 500 mg L^−1^ of phenol, (**C**) with 750 mg L^−1^ of phenol, (**D**) with 1000 mg L^−1^ of phenol. Numbers in the legend represent bacterial strains used in the experiment: 9—*K. oxytoca*, 11—*Lelliottia* sp., 14—*K. oxytoca*, 27—*S. marcescens*, 37—*H. alvei*, 43—*H. paralvei*, 51—*S. nematodiphila*. Negative control sample was represented by the duckweeds grown in sterile conditions (bacteria-free). Data represent the mean ± SE (*n* = 3).

Experiments with the long-term cultivation (14 days) of duckweeds showed that the multiplication of duckweeds in the presence of phenol (Figure 5) continued even at very high phenol concentrations, although the multiplication rate decreased as the concentration increased. Bacteria promoted the multiplication of duckweeds in the presence of phenol at all initial concentrations (Figure 5).

In the phenol-free MS medium with bacteria, a linear increase in the multiplication rate after seven days of cultivation was observed, whereas the sterile duckweed specimens (phenol-free, bacteria-free) stagnated (Figure 5A).

Initial phenol concentrations of 500 mg L^−1^ and 750 mg L^−1^ were associated with similar plant growth patterns during the first 7 days of the experiment, where, on average, phenol induced higher multiplication rates compared to phenol-free control (Figure 5B,C). After 7 days, however, phenol led to a gradual decrease in multiplication rates with the exception of sterile duckweeds at 500 mg L^−1^ of phenol (Figure 5B) and duckweeds co-cultivated with strain 11 (Figure 5C). An extremely high level of phenol (1000 mg L^−1^) decreased the multiplication rates of duckweeds (Figure 5D).

When it comes to the bacterial influence in the phenol-supplemented media, the *K. oxytoca* strain 14 induced a dramatic increase in the duckweed multiplication rate in the medium with 500 mg L^−1^ of phenol (Figure 5B) in the first week. After the 7th day, it also led to a rapid wilting and decay of the plants.

**Figure 6:**
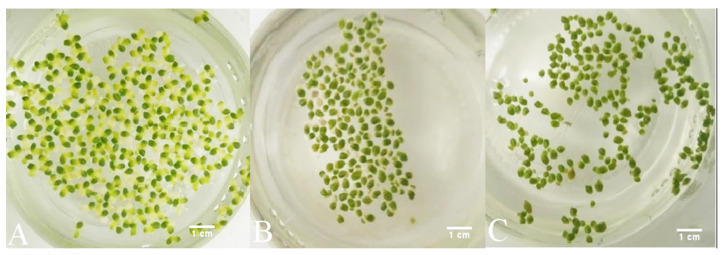
Macroscopic changes of duckweeds at high phenol concentrations: (**A**) at 500 mg L^−1^, chlorosis is extensive; (**B**) at 750 mg L^-1^ chlorosis is observed, but to a much lesser extent than at 500 mg L^−1^ (**A**); (**C**) at 1000 mg L^−1^, chlorosis is almost completely absent and the fronds appear to be darker green and smaller than at 750 mg L^−1^ (**B**) and at 500 mg L^−1^ of phenol (**A**).

It is worth noting that all samples of duckweed exhibited chlorosis at 500 mg L^−1^, but not at 750 and 1000 mg L^−1^ of phenol. Macroscopically, the fronds at 500 mg L^−1^ appeared larger (approx. 2 mm in diameter) and light green with approx. 50% of fronds affected by chlorosis. At 750 and 1000 mg L^−1^, they remained relatively small (approx. 1.5 mm in diameter) and dark green with less than 10% of fronds affected by chlorosis at 750 mg L^−1^ of phenol and less than 5% of fronds affected by chlorosis at 1000 mg L^−1^ (Figure 6).

The interesting behavior of co-cultivated bacteria and plants in the medium with 500 mg L^−1^ prompted further investigations regarding phenol-removal with this particular initial concentration.

### 2.5. Phenol Removal from the Solution by Bacterial Monocultures, by Duckweed, and by the Bacteria/Duckweed System

After five days of the experiment, the flasks containing bacterial monocultures (Figure 5A) contained between 9 mg L^−1^ of phenol (flask containing strain 14) and 110 mg L^−1^ of phenol (flask containing strain 27), while at the same time, in the control sample, the final concentration of phenol was approx. 363 mg L^−1^. As phenol is a volatile and relatively photosensitive substance, these processes probably contributed to the loss of phenol over time: in control flasks (sterile MS solution supplemented with phenol), there was a decrease of approx. 28% from the initial concentration of phenol.

**Figure 7:**
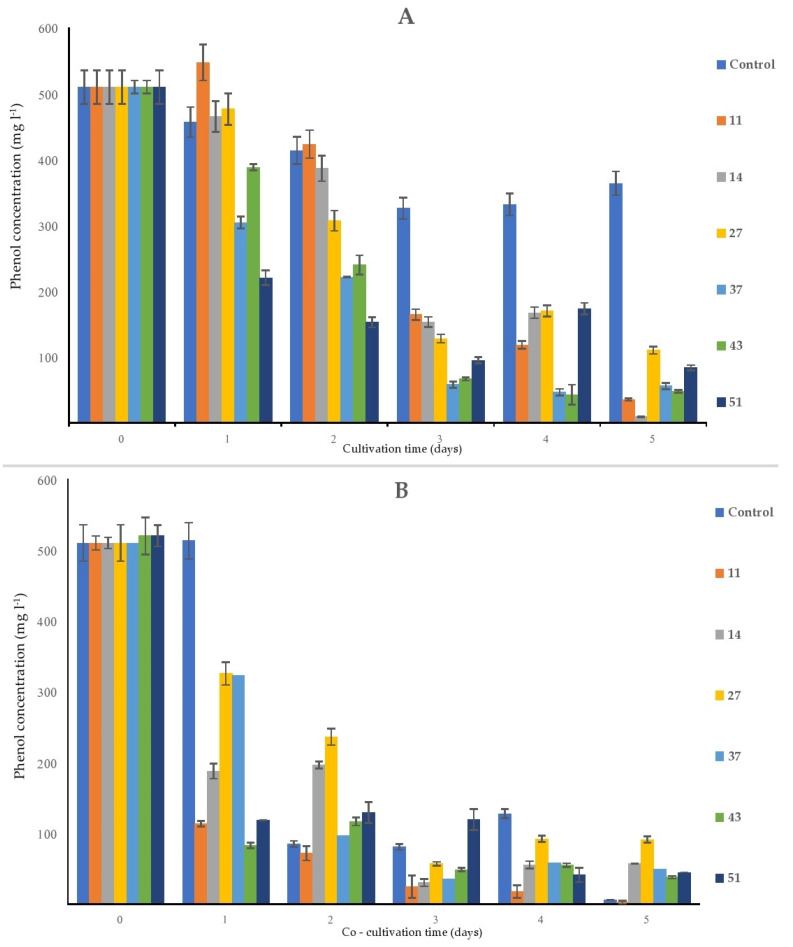
Removal of the initial phenol (500 mg L^−1^) by the bacterial strains alone (**A**) and by bacteria and duckweed combined (**B**). Negative control is (**A**) a sterile MS solution with phenol; (**B**) sterile duckweeds in an MS solution with phenol. Numbers in the legend represent bacterial strains used in the experiment: 9—*K. oxytoca*, 11—*Lelliottia* sp., 14—*K. oxytoca*, 27—*S. marcescens*, 37—*H. alvei*, 43—*H. paralvei*, 51—*S. nematodiphila*. Data represent the mean ± SE (*n* = 3).

In the flasks containing only sterile duckweed specimens, phenol removal was practically negligible after 1 day of cultivation. Between days 1 and 2, a steep decrease in the phenol content (approx. 83%) occurred in flasks with only surface-sterilized duckweeds (Figure 7B).

The most consistent decrease of the initial phenol concentration was detected in the MS medium that contained *H. paralvei* (strain 43) and duckweed: from 500 to approx. 83 mg L^−1^ after only one day of cultivation, after which the phenol content in these flasks slowly declined until it reached approx. 38 mg L^−1^ at the end of the experiment (Figure 7B). The least efficient bacteria/duckweed system was the one with strain 27—after 5 days of the experiment, the final concentration of phenol was approx. 91 mg L^−1^.

## 3. Discussion

The strains used in this study belong to species known for their ability to form biofilms: most notably, *Hafnia* sp, *Serratia* sp, and *Klebsiella oxytoca* [30,31,32]. As expected, it was two strains of Hafnia, namely, *H. alvei* strain 37 and *H. paralvei* strain 43, that had the best ability to form a biofilm, which is in concordance with the relevant scientific literature concerning genetically close strains [30].

There is a scientific consensus that bacteria tend to adhere to any available immobile surface, organic or inorganic, and form biofilms, and that this process is often stimulated in the presence of various noxious agents [33]. Moreover, in the recent bioremediation studies, it is becoming increasingly clear that the biological “platforms” are advantageous to the inorganic surfaces as a source of nutrients and a physical barrier, which simultaneously enhances the PGP properties of the bacteria [34]. In this study, we observed bacterial microcolonies in the intercellular crevices between epidermal root cells, which is a form of bacterial organization known to correspond to the early stages of biofilm formation [35]. The root exudates tend to leak from these intercellular spaces, therefore attracting the bacteria, which subsequently tend to organize into microcolonies and fully-formed biofilms [21,28,35]. This is a property that is reported to be useful in very different bioremediation strategies [25,35].

Since all the bacterial strains isolated from the rhizosphere of *L. minor* represent commensal microbes and opportunistic pathogens, and since the rhizosphere is an area of intense horizontal gene transfer through which antibiotic resistance is acquired, there is a concern about their re-introduction into the environment for bioremediation purposes [36]. Clinical isolates of *K. oxytoca*, *S. marcescens*, and *Lelliottia* sp., in particular, tend to possess resistance to one or more antibiotics, and multi-drug resistance has also been reported [37,38,39,40]. Clinical specimens of *H. alvei* and *H. paralvei* show more susceptibility to various antibiotics than the aforementioned species [40]. Unlike clinical specimens, all environmental bacterial strains tested in this study were susceptible to all major groups of antibiotics, which implies that these strains are safe and manageable for the potential application in bioremediation.

The fact that both *Klebsiella oxytoca* strains used in this study (strain 9 and 14) formed biofilms in vitro, but only one (strain 14) formed intimate connections with the root system of duckweed, implied a higher level of biological specificity of the tested bacteria–plant interactions, which is also supported by similar studies [32,34].

In the phenol-supplemented media, all bacteria shifted from their bacillary form to a coccoid one. This comes as no surprise since multiple bacterial species can change their morphology from bacillary to coccoid in response to various external stimuli [41].

Micrographs containing *K. oxytoca* (strain 14) suggest that this strain inhabited the interior of the cell, as is the case with multiple identified non-clinical isolates of *K. oxytoca*, which are described as endophytes [34]. This trait explains, at least in part, the phenol removal efficiency of the strain (the plant provides both biological and mechanical protection to the endophytic bacteria) and the positive effect on multiplication rates of duckweed. A similar effect was described in detail by other authors [42].

The structural damage caused by phenol was detected on the root system of *Lactuca sativa* in our previous study [43]. Similar effects were observed on the roots of the duckweed specimens: the cell wall thickening and the abundance of plasmids were associated with an increased lignin synthesis, a common stress response in plants [44].

It is worth noting that, due to their responsiveness to the environmental perturbances, many Lemnaceae are being used as bioindicators in ecotoxicological assays where the growth inhibition and pigment content are taken as common criteria for the assessment of toxicity [16]. The increase in the chlorophylls and carotenoids content is apparently common for duckweed specimens exposed to high phenol concentrations, as reported by Basiglini et al. [45]. This explained the dark green pigmentation manifesting in our specimens only when exposed to extremely high concentrations of phenol (750 and 1000 mg L^−1^). Similarly, the increased chlorophyll content was detected in our previous study with the *L. sativa* root system [43]. The apparent increase in chlorophyll content in duckweeds exposed to 750 and 1000 mg L^−1^ of phenol as compared to duckweed exposed to 500 mg L^−1^ of phenol is explained by plants’ anti-oxidant response. On the other hand, 500 mg L^−1^ of phenol is a signal to plants to propagate via multiplication and thus ensure the survival of the population but at the expense of more damage on chloroplasts (hence the observed chlorosis at 500 mg L^−1^ and not at 750 and 1000 mg L^−1^) [46].

None of the bacterial strains exerted a negative influence on the plants under basal conditions without phenol. On the contrary, all seven bacterial strains positively affected the multiplication rates of the duckweed specimens (the sole exception is the *K. oxytoca* strain 14 in the medium with 500 mg L^−1^ of phenol). The strains used in this study are probably not IAA producers; however, the limitation of Salkowski essay applied in this study implied that tested strains might still have produced a low-level of IAA sufficient to affect the growth of duckweed [29,47]. The interactions of IAA-producing bacteria and aquatic plants are further complicated by the fact that it only recently emerged that exogenous IAA probably affects the growth of aquatic plants, Lemnaceae included, in a completely opposite way to the terrestrial plants that were used as a model in the majority of PGP studies [47].

If tested strains were indeed not PGP, the positive effect on the multiplication rates is easily explained by dead bacteria being an additional nutrient source for the plants, especially an additional source of nitrogen and phosphorus, which are the greatest limiting factors for plant growth [48]. This is of particular importance in the bioremediation of oligotrophic aquatic ecosystems [24,28].

To ascertain if any of the tested bacterial strains were truly plant-growth promoting, additional analyses are necessary. For instance, PGP *A. calcoaceticus* P23 is a gibberellin producer and a phosphorus-solubilizer with visible PGP effects on *L. aoukikusa*, a species very similar to *L. minor* [25]. However, these analyses are beyond the scope of the current study.

The bacterial strains applied in this research were able to eliminate more than 80% of the initial phenol after 5 days, acting on their own. Furthermore, when combined with duckweed, this efficiency neared 100% after 5 days of co-cultivation. We detected a delayed removal of phenol by the surface-sterilized plants—a steep decrease in phenol content was detected after the second day of cultivation, whereas the bacteria and bacteria combined with duckweed showed a detectable decrease in phenol content after 1 day. A higher phenolic content at the end of the experiment in flasks with duckweed and bacteria was probably attributable to plant-bacteria signaling via phenolic compounds. However, since this was a short-term phenol removal experiment, it would be interesting to investigate whether the phenolic content changes in the long-term. This short-term experiment showed that the bacteria accelerated phenol removal during the first day of co-cultivation compared to surface-sterilized duckweed. Until relatively recently, phenol-degrading bacteria were isolated from contaminated soil samples or industrial wastewaters with very high phenol content [6,20,26,27]. The fact that the rhizosphere of certain plants can also be the source of phenol-degrading bacteria is a relatively recent discovery, and even less is known about the rhizosphere of aquatic plants [22,24]. Therefore, this study expands the list of phenol-degrading bacteria from the duckweed rhizosphere. Although the 4-AAP method applied in this study does not discriminate between phenolic compounds, exogenous and endogenous, according to our previous results, the amount of phenols synthesized by the plants and the microbial community under similar experimental conditions was negligible [49]. Therefore, it is our belief that the interference with the measurement is minimal.

Our results regarding the phenol removal presented in this study pointed at *L. minor* as a promising bioremediation agent in comparison to some other Lemnaceae species. For instance, *Spirodela polyrhiza* cannot eliminate phenol without bacteria from its own rhizosphere at all [27]. Similarly, phenol removal by sterile *L. aoukikusa* is modest but becomes enhanced when inoculated with *A. calcoaceticus* P23 [25]. It seems that this property of *L. minor* also surpasses the ability of some other, unrelated species to eliminate phenol. For example, lettuce and its transformed roots eliminated 200 or 100 mg L^−1^ after ten days [43]. In this case, sterile duckweed specimens practically achieved 100% of phenol removal after merely five days.

To the best of our knowledge, no research has so far addressed the ability of *Hafnia* sp. to eliminate phenol or any other organic pollutant. Regarding bioremediation, *Hafnia* sp. is only described in the context of the accumulation of nickel [50]. In our previous study, we concluded that *H. alvei* and *H. paralvei* strains possessed a remarkably high resistance to phenol and an ability to grow on minimal medium with phenol as the sole carbon source [51]. This current study expanded these results and confirmed that *H. alvei* and *H. paralvei* indeed eliminate phenol, in addition to being able to colonize the root surface, indicating a more intimate association with the plant compared to, for example, *K. oxytoca*, strain 9, which apparently has no affinity for the root surface whatsoever.

## 4. Materials and Methods

### 4.1. Plant Material and Cultivation Conditions

Mature duckweed specimens (*L. minor* L.) were collected from a pond in the garden of the Institute for Biological Research “Siniša Stanković”, the University of Belgrade (44°47’13.9092’’ N, 20°27’26.1828’’ E). Plants were washed with tap water for 20 min and then immediately surface-sterilized for 5 min in a commercial bleach solution containing 5% (*v/v*) sodium hypochlorite. The excess bleach was washed off with sterile distilled water three times. Plants (2–4 fronds) were placed on a Murashige-Skoog (MS) medium without agar (liquid medium) according to the previously applied protocol [49]. Plants were grown at 24 ± 2 °C (under fluorescent light of 40 μmoL m^−2^ s^−1^ with 16 h light/8 h dark photoperiod).

### 4.2. Bacterial Cultures and Cultivation Conditions

Bacterial cultures were grown and maintained on a Luria–Bertani (LB) medium, prepared according to the previously applied protocol [49,51]. Bacterial strains used in this experiment were as follows: strain 9—*Klebsiella oxytoca* A6-104/2, MF526910; strain 11—*Lelliottia* sp. 11bg, MK212916; strain 14—*Klebsiella oxytoca* 14bg, MK212915; strain 27—*Serratia* sp. A7-102/1, MF526924; strain 37—*Hafnia alvei* C31-106/2, MF526934; strain 43—*Hafnia paralvei* C32-106/3, MF526939; strain 51—*Serratia nematodiphila* D1-104/1, MF526945. Strains 9, 11, 14, 27, and 51 were found to be intimately connected with the root, whereas strains 37 and 43 were isolated from water in the proximity of the root, as reported in our previous study [51].

Additionally, these strains were selected owing to their phenol-resistance and the ability to grow on a minimal medium with phenol as the sole carbon source (also reported in our previous research) [51].

### 4.3. Biofilm formation

The biofilm formation was tested in accordance with O’Toole et al. [33]. The samples of overnight cultures in the LB broth and MS medium with phenol (200 mg L^−1^) as the sole carbon source were placed in a 96-well microplate. This concentration of phenol (200 mg L^−1^) was used in our previous study to test bacterial resistance to phenol. According to the results of our previous study, the majority of tested rhizospheric bacterial strains were able to grow at this concentration [51]. The control sample was defined as a sterile medium (either MS or LB). LB was used as the reference medium, while MS with phenol as the sole carbon source was used to test biofilm formation under unfavorable conditions (and whether biofilm formation was promoted in MS medium with phenol). After the cultivation at 25 °C for 48 h, the medium was removed from the plate. The surface of the biofilm was rinsed with distilled water and stained with 0.1% (*w/v*) crystal violet (CV) solution overnight. The CV solution was removed, and the plate was washed with distilled water. The color rings, originating from the CV, were photographed. The CV absorbed by the biofilm was dissolved in a 30% solution of glacial acetic acid and quantified by measuring the absorbance at 590 nm. Every data point is the result of an average of three measurements.

### 4.4. Minimal Inhibitory Concentration (MIC) of Antibiotics

To test the safety of selected bacterial strains for potential application in bioremediation and their potential antibiotic resistance, MIC was determined for five typical, commercially available antibiotics: Marocen (fluoroquinolone), Mipecid (imipenem), Aminocen (aminoglycoside), Piptaz (piperacillin), and Cefepim (4th generation cephalosporine). Antibiotics were dissolved in a 0.7% LB agar with varying testing concentration ranges of antibiotics. The 0.7% LB agar was autoclaved and then cooled to approx. 45 °C. The control was set as the soft LB agar without antibiotics. Next, 10 µL of 0.5 × 10^6^ CFU mL^−1^ was dropped on the top of the soft LB agar with and without antibiotics. The plates were visually inspected after 24 and 48 h of incubation. All plates were photographed. The concentration ranges of the antibiotics tested were selected according to the Performance Standards of Antimicrobial Susceptibility Testing (Control and Lab. Institute, 25th International Supplement, 2015); the same guidelines were used to interpret the minimal inhibitory concentration (MIC) of each antibiotic [52].

### 4.5. Fluorescence Microscopy

After the co-cultivation of duckweed with individual bacterial strains on the liquid MS medium with 200 mg L^−1^ of phenol for five days, the plants were collected. Fluorescent staining was performed with the Acridine Orange (AO) fluorescent dye, in accordance with the Kronvall and Myhre staining protocol [53]. Namely, individual duckweed specimens were immersed in the 0.01% solution of the AO (pH 3.0). After 2 min, the excess dye was rinsed in double-distilled water. The excised roots were placed on a microscopic slide, mounted in water/glycerol mixture, and coverslipped. The coverslip was gently pressed in order to flatten the tissue. The slides were sealed with clear nail polish and examined under an Axio Vert (Carl Zeiss, Germany) fluorescence microscope, in the excitation range of 450–500 nm and the detection range of 515–565 nm, with 40×, 63×, and 100× magnification, respectively. Filter used was FITC: Chroma set 41001, Excitation 460–500 nm, Emission 510–560 nm (bandpass filter).

### 4.6. Salkowski Reagent Test for the Colorimetric Indol-3-Acetic Acid (IAA) Detection

Following the observation that bacterial presence increased the multiplication rates of duckweed plants, all bacterial strains were tested for the production of IAA in the liquid MS medium in accordance with Gordon and Weber (1951), with some modifications [29,47], to test whether bacterial IAA might be the cause of this increase. Bacterial cultures were grown for 5 days under non-shaking conditions in 100 mL of the MS medium. Subsequently, 1 mL of bacterial suspension was centrifuged at 11,200× *g*. The supernatant was transferred to 1.5-mL Eppendorf tubes and mixed with 2 mL of Salkowski reagent (2% of 0.5 M FeCl_3_ in 35% HClO_4_ solution). The samples were incubated in the dark for 30 min. The IAA production in the cultured medium is indicated by the formation of pinkish to red coloration. The quantification is colorimetric and is conducted at OD 530 nm.

### 4.7. Multiplication Rate of Duckweed with or without Bacteria

Sterile plants (150 ± 20 fronds) were placed in flat-bottomed glass flasks containing 100 mL of the liquid MS medium supplemented with 500, 750, and 1000 mg L^−1^ of phenol and in the flasks containing MS liquid medium without phenol (Figure S1). This concentration range of phenol was used in our previous study to test bacterial phenol-resistance and was used again in this study to test the resistance of plants. Subsequently, the media were inoculated with appropriate bacterial strains (final dilution: 0.5 × 10^5^ CFU mL^−1^). The control samples were bacteria-free. The duckweed specimens were cultivated for 14 days and photographed at selected time points. The number of newly formed fronds was estimated by ImageJ.

Multiplication rates will be calculated according to Equation (1):

(1) (Number of newly formed fronds)/(Initial number of fronds)

The results were presented graphically, as an average of at least two consecutive experiments.

### 4.8. Phenol Removal Test

Sterile duckweed cultures were used as the basic material for the phenol removal test. Approximately 150 individual duckweed specimens were initially grown on the MS medium supplemented with 500 mg L^−1^ of phenol. Individual bacterial monocultures and plants with a single bacterial monoculture in the same flask were grown at the same phenol concentrations. The final dilutions of bacterial suspensions in the MS media were 0.5 × 10^5^ CFU mL^−1^ for co-cultivation with duckweed and 1 × 10^5^ CFU mL^−1^ in the samples with bacteria alone. The flasks were incubated at 30 °C on a rotation shaker at 150× *g* for 24 h.

#### Determination of Phenol Concentration in the MS Solution

The determination of the phenol concentration in the solution was done spectrophotometrically using 4-aminoantipyrine (4-AAP). The samples were treated with phosphoric acid to pH 4.0 and then distilled prior to the spectrophotometric measurement.

The mixture prepared for the measurement of the phenol concentration in water contained the distilled sample of the medium, buffer (16.9 g ammonium chloride dissolved in 143 mL of ammonium hydroxide, pH 10), 2 mL of the 2% (*w/v*) 4-AAP solution and 2 mL of the 80% (*w/v*) potassium ferricyanide solution. The reaction solution was mixed, and, after 15 min, absorbance was read at 460 nm (maximal absorption of the compound formed in the reaction of 4-AAP). The absorbance of the standards was monitored at the same wavelength, and the dilutions of phenol were prepared in the concentration range from 0 to 1000 μg L^−1^. All measurements were made according to the regulations of the Institute for Standardization of Serbia, the ISO code 6439 B: 1997, and the water quality-determination of phenol index (Institute MOL, Stara Pazova, Serbia).

### 4.9. Statistical and Image Analysis

The numerical data were analyzed using R (R Core Team, 2017) [54]. Each value represents the mean ± standard error (SE). All graphs were generated by Microsoft Excel. Microscopic photographs were generated by AxioVision Software, version 4.8.

## 5. Conclusions

In this study, we demonstrate a beneficial interaction between the common duckweed *L. minor* and selected bacteria from its rhizosphere. The results suggest that a dual bacteria/duckweed system is more useful in bioremediation than the plants or the bacteria alone. Other possible advantages of this system are also reported: the bacteria provide an additional nutrient source for the plants and accelerate their multiplication rates. Biofilm-bound bacteria can be removed with the plants after the bioremediation is finished. Fluorescence microscopy revealed that the bacteria were most abundant in the middle portion of the root and that they formed microcolonies in the intercellular crevices between the epidermal root cells. Moreover, the selected bacterial strains were found to be sensitive to typical antibiotics and hence safe for potential use in bioremediation. The combination of *H. paralvei*, strain 43, and duckweeds was the most promising bioremediation platform due to the accelerated removal of phenol and multiplication rates of the duckweeds. There is a possibility that all applied bacterial strains are also PGP bacteria, which would make them applicable in other areas besides bioremediation (e.g., agriculture). However, more studies are needed before such a conclusion can be drawn.

## Supplementary Materials

The following are available online at https://www.mdpi.com/2223-7747/9/5/599/s1, Figure S1: Flasks with bacterial strains and duckweed in MS nutrient medium after 14 days of cultivation, Table S1: Results of 16S rDNA sequencing for previously taxonomically undetermined bacterial strains (11 and 14) and of gyrB sequencing for *Serratia* sp. (27) and *Serratia nematodiphila* (51), and Table S2: MIC of antibiotics.
