# Supplementary material for: Phenol Removal Capacity of the Common Duckweed (Lemna minor L.) and Six Phenol-Resistant Bacterial Strains From Its Rhizosphere: In Vitro Evaluation at High Phenol Concentrations
Source: Plants (Basel). 2020 May 8;9(5):599. doi: 10.3390/plants9050599 (PMC7285011; doi:10.3390/plants9050599)
Supplement: Supplementary file 1 [file plants-09-00599-s001.pdf]

## 11. Supplementary Material:

### 11.1. DNA extraction, gyrase B and 16S rRNA genes amplification

DNA of the strains of interest was extracted according to Le Marrec et al. (2000), with some modifications. Primers used for amplification of gyrase B gene segment are universal for Gram-negative bacteria: UP-1S (GAAGTCATCATGACCGTTCTGCA: forward primer) and UP-2Sr (AGCAGGGTACGGATGTGCGAGCC: reverse primer), which cover a segment of approximately 1200 base pairs. Primers for the 16S ribosomal RNA gene segment were as follow: UN1-16SF (GAGAGTTTGATCCTGGC) and UN1-16SR (AGGAGGTGATCCAGCCG) and cover a segment of 1492 base pairs. The quality of DNA extracts was checked in 1% agarose gel after 1 hour run at 90 volts. Resulting bands were visualized under UV light. DNA was amplified in a reaction volume of 28 μl which contained 10× KAPA Taq Buffer, 2.4 μl 25 mM magnesium(II)chloride, 0.6 μl 10mM nucleotide mix, 1.2 μl of 10 μM primers, 1.5 μl of 5U per 1μl of KAPA Taq DNA Polymerase (KAPA Biosystems, Boston, USA). The PCR products were analyzed in 1% agarose gel with 4 μl of Ethidium-Bromide under the same conditions, visualized with UV light and cut from the gel. Gel cut-outs were purified with Gel Purification Kit (Qiagen, Germany) according to the manufacturer's instructions. Purified gel samples were subsequently sent to Macrogen, the Netherlands for Sanger dideoxy sequencing.

### 11.2. 16S rRNA and gyrB sequencing

Previously isolated strains (11, 14bg, 27, 51) were identified according to the results of 16S rRNA and gyrB sequencing, respectively (Table S1). Only the optimal BLAST

results were taken into consideration. Strains 11 and 14 were assigned with appropriate 16S rRNA sequences. The gyrB sequences of strains 27 and 51 suggest that they are both more closely related to *S. marcescens*.

Table S1. Results of 16S rDNA sequencing for previously taxonomically undetermined bacterial strains (11 and 14) and of gyrB sequencing for *Serratia sp.* (27) and *Serratia nematodiphila* (51).

| Sequence | Closest relative | Identity | ID of the closest relative |
|---|---|---|---|
| 27 gyrB | *Serratia marcescens* SSM2560 | 96% | DQ386886 |
| 51 gyrB | *Serratia marcescens* RSC-14 | 93% | CP012639 |
| 11 16S rRNA | *Lelliottia sp.* GL2 | 95% | KM458060 |
| 14bg 16S rRNA | *Klebsiella oxytoca* LF-1 | 96% | EF127829 |

### 11.3. Minimal inhibitory concentration (MIC) of antibiotics

Five typical, commercially available antibiotics: Marocen (Fluoroquinolone), Mipecid (Cilastatin + Imipenem), Aminocen (Aminoglycoside), Piptaz (Piperacilline), and Cefepim (4$^{th}$ generation Cephalosporine) were dissolved in a 0.7% LB agar with varying testing concentration ranges of antibiotics. The 0.7% LB agar was autoclaved and then cooled to approx. 45 °C. The control was set as the soft LB agar without antibiotics. Next, 10 μl of $0.5\times10^{6}$ CFU $ml^{-1}$ was dropped on the top of the soft LB agar with and without antibiotic. The plates were visually inspected after 24h and 48h of incubation. All plates were photographed.

The concentration ranges of the antibiotics tested were selected according to the Performance Standards of Antimicrobial Susceptibility Testing (Control and Lab. Standards Institute, 25th International Supplement, 2015); the same guidelines were used to interpret the Minimal Inhibitory Concentration (MIC) of each antibiotic.

Table S2. MIC of antibiotics.

| Bacterial strains: | MIC (mg $l^{-1}$) of an antibiotic: | | | | |
|---|---|---|---|---|---|
| | **Aminocen (Aminoglycoside)** | **Piptaz (Piperacilline)** | **Cefepim (4th generation Cephalosporine)** | **Mipecid (Cilastatin + Imipenem)** | **Marocen (Fluoroquinolone)** |
| *Klebsiella oxytoca (9)* | 8 | 2 | 0.13 | 0.13 | 0.13 |
| *Lelliottia sp. (11)* | 4 | 2 | 0.13 | 0.06 | 0.13 |
| *K. oxytoca (14)* | 4 | 2 | 0.06 | 0.06 | 0.06 |
| *Serratia marcescens (27)* | 8 | 2 | 0.25 | 0.06 | 0.13 |
| *Hafnia alvei (37)* | 8 | 2 | 0.13 | 0.13 | 0.13 |
| *H. paralvei (43)* | 8 | 2 | 0.13 | 0.06 | 0.06 |
| *S. nematodiphila (51)* | 8 | 2 | 0.13 | 0.13 | 0.13 |

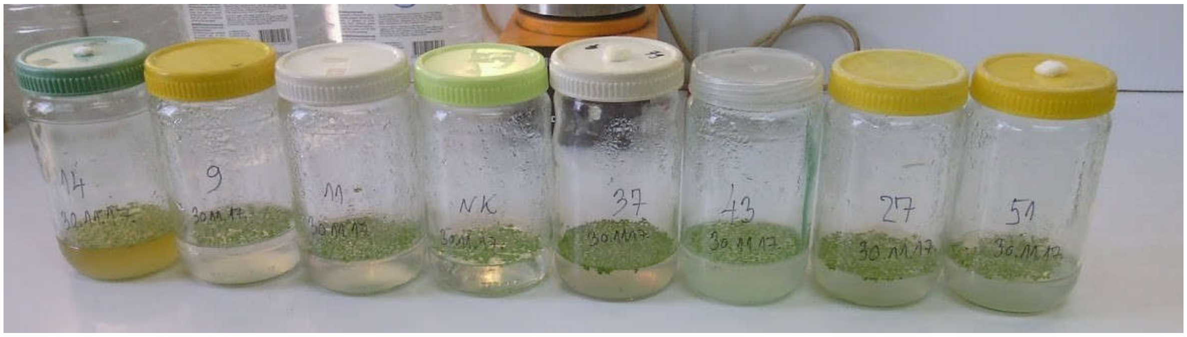

Fig. S1. Flasks with bacterial strains and duckweed in MS nutrient medium after 14 days of cultivation: from left to right: *K. oxytoca*, strain 14 – when co-cultivated with duckweed, only this strain stained the nutrient medium, both with and without phenol which is probably due to endophytic lifestyle of this bacterium; *K. oxytoca*, strain 9; *Lelliottia* sp. strain 11; NK – negative control (surface-sterilized duckweed); *H. alvei*, strain 37; *H. paralvei*, strain 43; *S. marcescens*, strain 27; *S. nematodiphila*, strain 51).
